# Keratinolytic Properties of *Aspergillus clavatus* Promising for Biodegradation

**DOI:** 10.3390/ijerph192113939

**Published:** 2022-10-26

**Authors:** Svetlana Timorshina, Elizaveta Popova, Valeriana Kreyer, Nina Baranova, Alexander Osmolovskiy

**Affiliations:** Department of Microbiology, Faculty of Biology, Lomonosov Moscow State University, 119991 Moscow, Russia

**Keywords:** *Aspergillus clavatus*, fungal proteases, micromycetes, keratinases, keratin waste, biodegradation

## Abstract

The high demand for keratinolytic enzymes and the modest presentation of fungal keratinase diversity studies in scientific sources cause a significant interest in identifying new fungal strains of keratinase producers, isolating new enzymes and studying their properties. Four out of the 32 cultures showed a promising target activity on protein-containing agar plates—*Aspergillus amstelodami* A6, *A. clavatus* VKPM F-1593, *A. ochraceus* 247, and *Cladosporium sphaerospermum* 1779. The highest values of keratinolytic activity were demonstrated by extracellular proteins synthesized by *Aspergillus clavatus* VKPM F-1593 cultivated under submerged conditions on a medium containing milled chicken feathers. The enzyme complex preparation was obtained by protein precipitation from the culture liquid with ammonium sulfate, subsequent dialysis, and lyophilization. The fraction of a pure enzyme with keratinolytic activity (pI 9.3) was isolated by separating the extracellular proteins of *A. clavatus* VKPM F-1593 via isoelectric focusing. The studied keratinase was an alkaline subtilisin-like non-glycosylated protease active over a wide pH range with optimum keratinolysis at pH 8 and 50 °C.

## 1. Introduction

The food demand of the humanity is increasing due to the global population growth, thereby increasing the environmental burden. Already in 2019, the production of chicken meat exceeded 115 million tons [1], while poultry farming is one of the main agricultural sectors, which results in the formation of keratin-rich waste [2]. Keratins are a group of hard-to-degrade fibrous proteins that play a key role in the formation of the epidermis and its derivatives, such as wool, bristles, and feathers [3]. Currently, there are various approaches to animal byproduct disposal. Incineration and landfilling are the cheapest ones, however, they are harmful to nature and human health [4,5]. Other methods, e.g., acid or alkaline hydrolysis and carbonization, on the one hand, enable obtaining useful substances from organic waste—amino acids applied as feed additives and carbon materials for biocatalysis and bioremediation [6,7,8]. On the other hand, these processing techniques are energy-consuming, require application of caustic reagents and special equipment. Biodegradation is a sustainable way to dispose of waste and a perspective alternative to common approaches in this area. The application of microorganisms and their enzymes allows avoiding the formation of compounds harmful to humans and the environment; moreover, the resulting amino acids and oligopeptides can be used in agriculture, pharmaceuticals, and medicine [9,10]. Furthermore, proteases are biosafe agents and are neutralized by denaturation.

Keratinolytic enzymes have been actively investigated and described since the second half of the previous century and have extensive prospects for application not only in biodegradation, but also in other industrial sectors, for example, in the food and leather production [11,12]. It is noteworthy that the focus of scientific works on keratinases, which are mainly microbial metabolism products, stays on enzymes of bacterial origin [13,14,15,16,17,18,19,20]. This fact is obviously associated with a better investigation of these organisms as objects of scientific research and biotechnological processes in comparison with fungal models, although filamentous fungi are also capable of secreting hydrolases that destroy keratin [21]. Most of the accumulated knowledge about fungal keratinases relates to enzymes synthesized by dermatophytes [22,23,24,25,26,27,28,29], while data on keratinolytic enzymes of micromycetes that do not cause dermatophytosis remain scarce [9,21].

Nowadays, the capacity of filamentous fungi to secret proteases breaking down peptide bonds in fibrous proteins in a wide range of conditions, as well as the possibility of micromycete cultivation on agricultural waste, including systems with low water activity, make them encouraging candidates for keratinase producers [30,31]. In this regard, this work is devoted to investigating the ability of non-dermatophyte micromycetes belonging to the Ascomycota phylum to synthesize keratinolytic enzymes promising for biodegradation.

## 2. Materials and Methods

### 2.1. Materials

Chicken feathers and milled chicken feathers were bought at a local store. The chicken feathers for use were soaked in a 0.1% Tween 20 solution and vigorously stirred for 20 min, then washed with distilled water three times, and incubated at 87 °C until completely dried. The remaining chemicals and reagents were of analytical grade. Keratin from wool was purchased from Tokyo Chemical Industry Co., Ltd. (Tokyo, Japan). Salts were obtained from PanReac (Barcelona, Spain). Ampholines were purchased from Bio-Rad (Hercules, CA, USA). Pre-stained protein ladder was obtained from Thermo Scientific (Waltham, MA, USA). Reagents for electrophoresis and other chemicals were purchased from Merck (Darmstadt, Germany).

### 2.2. Storage of the Microorganisms

Fungal cultures were obtained from the Department of Microbiology and the Department of Mycology and Algology at Lomonosov Moscow State University. They were maintained at 28 °C in wort agar slant tubes and subcultured every month. The fungal inoculum for all the fermentative analyses was spore mass of 5–7-day-long cultures.

### 2.3. Fungal Strain Screening for the Ability to Secrete Keratinolytic Enzymes

The fungi were grown on semi-selective agar media (0.05% KH_2_PO_4_, 0.025% MgSO_4_, 0.5% peptone, 1.5% agar, 1% or 0.5% protein substrate); 1.0% Hammerstein casein or gelatin or 0.5% keratin was added to the media as the main source of carbon and nitrogen. Inoculation was carried out by injecting into the center of the Petri dish (90 mm). After 7 days of cultivation at 28 °C, nonhydrolyzed proteins were precipitated with a 10% trichloroacetic acid (TCA) solution, and diameters of the colonies and hydrolytic diameters (clear halo zones) were measured. Analysis of hydrolytic efficacy was performed by calculation of enzymatic indices (EI) as follows: EI = d_2_/d_1_, where d_1_—diameter of the colony (mm), d_2_—hydrolytic diameter (mm).

The fungi showing high activity on protein-containing agar plates were grown in 100 mL liquid media (750 mL shaker flasks) at 28 °C, 200 rpm, 7 days. Modified Czapek media (Table 1) (pH 5.5) were used for screening in submerged conditions.

Culture supernatants were collected on the 3rd day and the 7th day after inoculation for enzyme activity assays.

Genomic DNA of the fungal strain that demonstrated the highest level of keratinolytic activity was extracted using a DNEasy Plant Mini Kit (Qiagen, Germany) to confirm the taxonomic position of the strain. Molecular identification was performed by sequence analysis of the variable D1/D2 domain of large subunit (26S) ribosomal DNA and the β-tubulin (*benA*) gene. The D1/D2 domain of large subunit (LSU) rRNA was amplified with primers NL-1 (5′-GCATATCAATAAGCGGAGGAAAG-3′) and NL-4 (5′-GGTCCGTGTTTCAAGACGG-3′) [32], and primers Bt2a (5′-GGTAACCAAATCGGTGCTGCTTTC-3′) and Bt2b (5′-ACCCTCAGTGTAGTGACCCTTGGC-3′) [33] were used for β-tubulin (*benA*) gene amplification. The sequences were deposited to the NCBI Genbank database under the OK559552.1 (LSU rRNA) and OP254188 (*benA*) accession numbers. The sequences for phylogenetic analysis from the NCBI Genbank database (Table 2) were analyzed with MEGA v10.0 [34]. A phylogenetic tree based on concatenated sequences of the β-tubulin and LSU rRNA genes was constructed using the maximum likelihood method and the Tamura–Nei model (1000 bootstrap replicates).

### 2.4. Enzyme Production Optimization

The fungus with the highest keratinolytic activity based on the results of the second step of screening was cultivated in 100 mL liquid medium for biomass accumulation (6.7% wort, 2.0% glucose, 0.1% peptone, pH 5.5) in a shaker flask (750 mL) at 28 °C, 200 rpm, 2 days. Then, 3% of the produced biomass (*v*/*v*) were transferred into modified Czapek media with various sources of keratin for analysis of the extracellular enzyme accumulation dynamics (medium 4: 0.3% NaNO_3_, 0.1% K_2_HPO_4_, 0.05% MgSO_4_ × 7H_2_O, 0.05% KCl, 0.001% FeSO_4_ × 7H_2_O, 3.0% glucose, 0.5% fish flour hydrolysate (FFH), 0.5% keratin, pH 6.0; medium 5: 0.3% NaNO_3_, 0.1% K_2_HPO_4_, 0.05% MgSO_4_ × 7H_2_O, 0.05% KCl, 0.001% FeSO_4_ × 7H_2_O, 3.0% glucose, 0.5% FFH, 0.5% milled chicken feathers, pH 6.0; medium 6: 0.3% NaNO_3_, 0.1% K_2_HPO_4_, 0.05% MgSO_4_ × 7H_2_O, 0.05% KCl, 0.001% FeSO_4_ × 7H_2_O, 3.0% glucose, 0.5% FFH, 0.5% chicken feathers, pH 6.0). The cultivation conditions were the same (100 mL medium, 28 °C, 200 rpm). Culture supernatants were collected from the 2nd to the 7th day after inoculation for enzyme activity assays.

### 2.5. Enzyme Activity Assay

The caseinolytic activity of extracellular proteases was determined by the modified Anson–Hagihara method [35]. The reaction was carried out with 200 μL 1% Hammerstein casein solution in 0.1 M Tris HCl buffer (pH 8.2) and 100 μL sample. The reaction mixture was incubated at 37 °C for 10 min in a thermal shaker at 600 rpm. The reaction was stopped by adding 300 μL 10% TCA solution. Then, the samples were centrifuged for 5 min at 14,000 rpm prior to the measurement of absorbance at 275 nm using a spectrophotometer (BioSpectrometer Kinetic, Eppendorf, Hamburg, Germany). The keratinolytic activity was measured by a similar method: 1% keratin suspension was prepared in 0.05 M Tris HCl buffer (pH 8.2); the reaction was carried out for 30 min. Absorbance measurements were performed at 280 nm.

Furthermore, proteolytic activity was investigated using chromogenic peptide substrates para-nitroanilides (CPS) to clarify protease substrate specificity; 200 μL sample and 100 μL 0.05% CPS (0.05 M Tris HCl buffer, pH 8.2) were incubated for 5 min at 37 °C (600 rpm). The reaction was stopped by adding 200 μL 50% acetic acid. Absorbance measurements were performed at 405 nm.

One unit (U) of activity was defined as an increase in absorbance by 0.01 under the assay conditions.

### 2.6. Enzyme Isolation

A complex of extracellular proteins was precipitated with ammonium sulfate (608 g per 1 L culture supernatant). After 48 h incubation at 4 °C, the proteins were centrifuged at 3160× *g* (4 °C) for 45 min in an LMC-4200R centrifuge (Biosan, Latvia). The precipitate was dissolved in 0.01 M Tris HCl buffer (pH 8.2) and dialyzed against 0.005 M Tris HCl buffer (pH 8.2) for 24 h prior to re-centrifugation under similar conditions. The supernatant was used for isoelectric focusing with the Westerberg method in a pH gradient of ampholines and a 0–40% sucrose density in a 110 mL column at 800 V and 4 °C. After 36 h, the resulting mixture was fractionated in 1.5 mL. For each fraction, different parameters were measured: pH, protein content (spectrophotometrically at 280 nm), and enzymatic activity. The purified enzyme was analyzed by sodium dodecyl sulfate polyacrylamide gel electrophoresis (SDS-PAGE) using a discontinuous buffer system [36]. A separation gel (15%, *w*/*v*) and a stacking gel (5%, *w*/*v*) were used. The electrophoretic migration of the keratinolytic enzyme was compared with a pre-stained broad-range protein ladder (Thermo Scientific, USA) to detect the protein’s molecular weight after staining with Coomassie Brilliant Blue R-250.

### 2.7. Detection of Enzyme Glycosylation

To establish if an isolated keratinase has a carbohydrate component, a qualitative reaction was performed using periodic acid and the Schiff reagent by means of dot blotting on nitrocellulose membranes [37].

### 2.8. Effects of Protease Inhibitors on Enzyme Activity

To investigate the effects of different inhibitors on enzyme activity, purified keratinase was preincubated in solutions of different reagents (0.05 M Tris HCl buffer, pH 8.2) for 1 h at 25 °C. Proteolytic activity was determined as a percentage of residual activity relative to control. The enzyme was treated with the following protease inhibitors: phenylmethylsulfonyl fluoride (PMSF; 1.5 mM), sodium ethylenediaminetetraacetate (EDTA; 1 mM), p-chloromercuribenzoic acid (PCMB; 1 mM), N-p-tosyl-L-phenylalanine chloromethyl ketone (TPCK; 0.5 mM), N-tosyl-L-lysine chloromethyl ketone hydrochloride (TLCK; 0.5 mM), soybean trypsin inhibitor (SBTI; 0.5 mg/mL).

### 2.9. Effects of pH and Temperature on Enzyme Activity

The optimal pH for purified keratinase activity was investigated by performing the enzyme reaction with a mixture (1:1, *v*/*v*) of protease and 0.4 M universal buffer (sodium acetate/phosphate/borate buffer, pH 3.0–11.0). The remaining proteolytic activity was assayed as previously mentioned. To determine the enzyme’s pH stability, the mixture was preincubated for 2 h at 37 °C and 600 rpm prior to assay proteolytic activity. The residual activities were expressed as a percentage of enzyme activity under the same conditions without preincubation.

The optimum temperature of the isolated keratinase was analyzed over a temperature range of 25–65 °C. Thermostability of the enzyme was assayed by 2 h preincubation of the sample at various temperatures before carrying out reactions. The residual activities were expressed as a percentage of enzyme activity under the same conditions without preincubation. The relative and residual proteolytic activities were assayed as previously mentioned at pH 8.2.

All the experiments were repeated in triplicates, and the results obtained were presented as the means ± standard deviation.

## 3. Results

### 3.1. Screening for Fungi That Efficiently Produce Keratinolytic Enzymes

**Figure 1:**
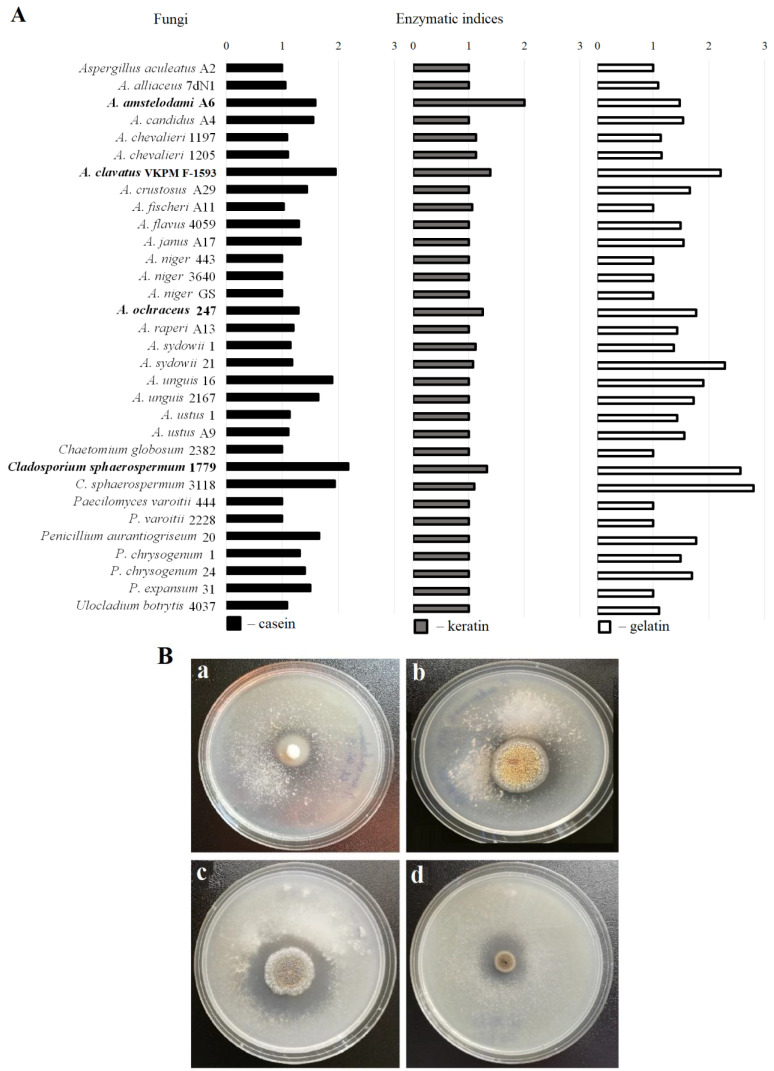
Screening for keratinolytic fungi on agar media. (**A**) Enzymatic indices (EI) of the studied strains grown on protein-containing agar plates. The media contained 1.0% casein (black), 0.5% keratin (grey), and 1.0% gelatin (white). The fungi in bold demonstrated the highest EI value on the keratin medium in combination with the presence of hydrolysis zones on the casein and gelatin media and were selected for further investigation. The experiment was done in triplicate. (**B**) Selected fungi on the casein agar medium after 5 days of cultivation: (**a**) *A. amstelodami* A6, (**b**) *A. ochraceus* 247, (**c**) *Aspergillus clavatus* VKPM F-1593, and (**d**) *Cladosporium sphaerospermum* 1779.

The proteolytic potential of 32 micromycete strains belonging to genera *Aspergillus*, *Chaetomium*, *Cladosporium*, *Paecilomyces*, *Penicillium*, and *Ulocladium* was evaluated using three agar media with different protein substrates: keratin, casein, and gelatin. The obtained data indicate that some representatives of the genera *Aspergillus*, *Cladosporium*, and *Penicillium* are active producers of extracellular proteases; however, only 10 out of the 32 cultures of microscopic fungi had an EI higher than 1 on the medium with keratin, namely *A. amstelodami* A6, *A. chevalieri* 1197 and 1205, *A. clavatus* VKPM F-1593, *A. fischeri* A11, *A. ochraceus* 247, *A. sydowii* 1 and 21, *C. sphaerospermum* 1779 and 3118 (Figure 1A). All the strains that effectively hydrolyze keratin also showed a high EI on the media with other protein substrates (Figure 1B).

The fungi that demonstrated significant keratinolytic activity on a solid medium (growth and activity ratio equal to or higher than 1.25) were selected to be tested on plates with media containing other fibrous proteins, such as fibrin and elastin. The *Aspergillus amstelodami* A6 strain had the highest ratio of the EI on the keratin medium to the EI on the casein medium. This strain also effectively hydrolyzed gelatin and fibrin. The lowest calculated coefficients on the media with keratin, fibrin, and elastin in relation to the EI on the casein medium belonged to *A. clavatus* VKPM F-1593 and *C. sphaerospermum* 1779 (Table 3).

**Figure 2:**
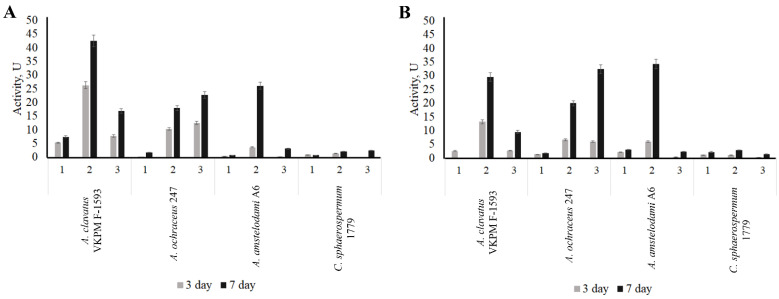
Keratinolytic (**A**) and caseinolytic (**B**) activity of selected fungi cultivated under submerged conditions on three different media: medium with wool keratin (1); medium with wool keratin and sodium nitrate (2); medium with sodium nitrate (3).

The next step of screening was carried out with four chosen cultures (*A. clavatus* VKPM F-1593, *A. amstelodami* A6, *A. ochraceus* 247, and *Cladosporium sphaerospermum* 1779) using liquid media. For submerged fermentation, three media differing in nitrogen sources were used. Medium 1 contained keratin, medium 2 was prepared with keratin and sodium nitrate, and sodium nitrate was the only source of nitrogen in medium 3. Caseinolytic and keratinolytic activities were measured in the culture liquid on the 3rd day and the 7th day of cultivation (Figure 2).

The *C. sphaerospermum* 1779 strain did not secrete extracellular enzymes with keratinolytic and caseinolytic activity under the experimental conditions. The maximum activity of micromycete *A. ochraceus* 247 (keratinolytic—22.7 U, caseinolytic—32.4 U) was observed using medium 3 for the microorganism growth. The addition of keratin to the medium reduced the accumulation of keratinases insignificantly, while the yield of caseinolytic enzymes decreased by 40% on the seventh day. The highest activity of the *A. clavatus* VKPM F-1593 and *A. amstelodami* A6 proteolytic enzymes was achieved when using a medium with two sources of nitrogen, keratin and sodium nitrate; on the seventh day of cultivation, the activity of *A. clavatus* VKPM F-1593 keratinases (42.5 E) exceeded the *A. amstelodami* A6 keratinolytic activity (26.1 U) by more than 1.5 times.

The highest keratinolytic activity values, both on the 3rd day and on the 7th day, were demonstrated by the *A. clavatus* VKPM F-1593 strain when performing submerged fermentation on medium 2. Thus, this culture was selected for further research.

**Figure 3:**
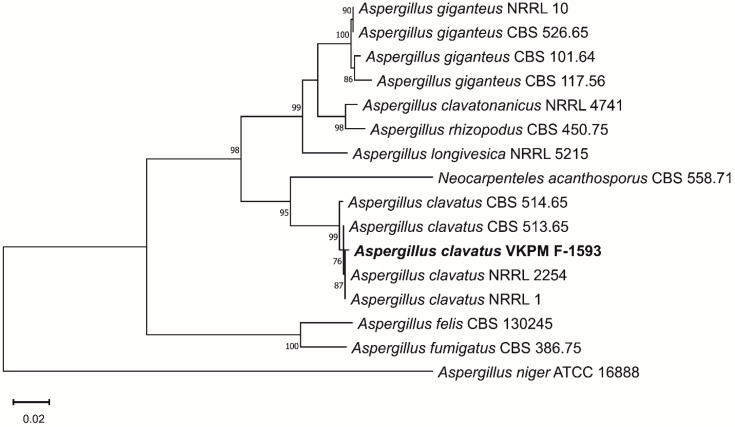
Phylogenetic dendrogram based on concatenated sequences of the *benA* and D1/D2 domain of the large subunit (26S) ribosomal RNA genes. DNA sequences of the *A. clavatus* VKPM F-1593 strain were compared with the strains belonging to *Aspergillus* section *Clavati* [38] and other closely related species. The phylogenetic tree was constructed by means of the maximum likelihood method with MEGA X ver. 10.0.5. Bootstrap analysis was performed using 1000 replicates. Substitutions per nucleotide position: 0.02. Only values above 70% are indicated.

Phylogenetic analysis was carried out to confirm the taxonomic position of the micromycete. The phylogenetic tree confirmed affiliation of the VKPM F-1593 strain with the clade of *Aspergillus clavatus* (Figure 3).

### 3.2. Improving the Enzyme Activity during Submerged Fermentation

**Figure 4:**
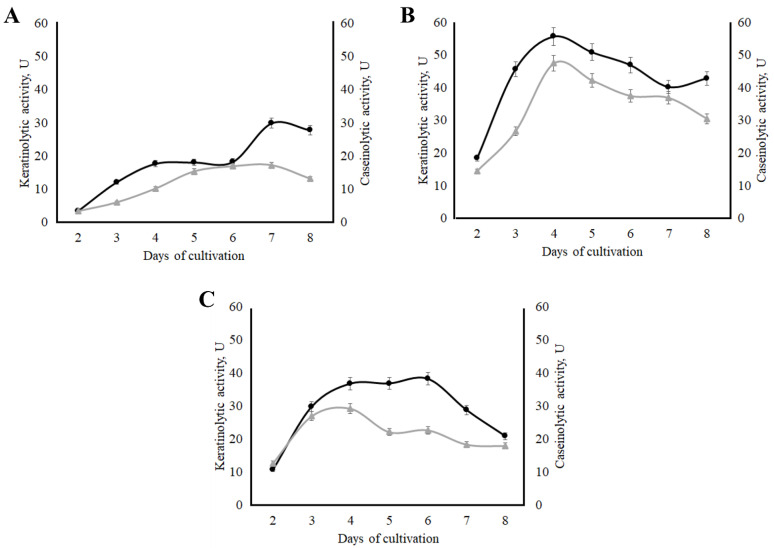
Accumulation of extracellular proteases synthesized by *A. clavatus* VKPM F-1593 during growth on the various media: (**A**) medium 4; (**B**) medium 5; (**C**) medium 6. Black lines—keratinolytic activity; grey lines—caseinolytic activity.

A two-stage submerged fermentation of the *Aspergillus clavatus* VKPM F-1593 strain was carried out on a medium rich in sugars to produce biomass, and then on media with various sources of keratin to obtain target enzymes. Medium 4 contained wool keratin, milled chicken feathers were added in medium 5, and medium 6 was prepared with whole chicken feathers. The assessment of extracellular proteolytic enzyme accumulation by micromycete *A. clavatus* VKPM F-1593 was registered from the 2nd to the 8th day of cultivation (Figure 4).

The highest absolute values of keratinolytic (55.6 U) and caseinolytic (47.5 U) activities were obtained when micromycete *A. clavatus* VKPM F-1593 was cultivated on medium 5. It is important to note that the peak proteolytic activity on this medium occurred on the 4th day of cultivation. On medium 6, the maximum keratinase accumulation (about 37.4 U) occurred in the time interval from the 4th to the 6th day of cultivation, and the highest caseinolytic activity (29.3 U) was on the 4th day. When *A. clavatus* VKPM F-1593 was cultivated on medium 4, the maximum activity of keratinases (29.9 U) occurred on the 7th day, and the highest caseinolytic activity (16.5 U) reached a plateau from the 5th to the 7th day.

In addition, alkalization of the medium was observed during submerged fermentation of the producer. The initial pH value of the fermentation medium was 6.0; already by the second day of micromycete growth on the medium with milled chicken feathers, the pH value of the culture liquid reached 7.5, and it was 8.8 on the 7th day.

Medium 5 was used for the further research of extracellular keratinolytic enzymes synthesized by *Aspergillus clavatus* VKPM F-1593.

### 3.3. Isolation of the Aspergillus Clavatus VKPM F-1593 Extracellular Keratinase and Investigation of Some of Its Properties

**Figure 5:**
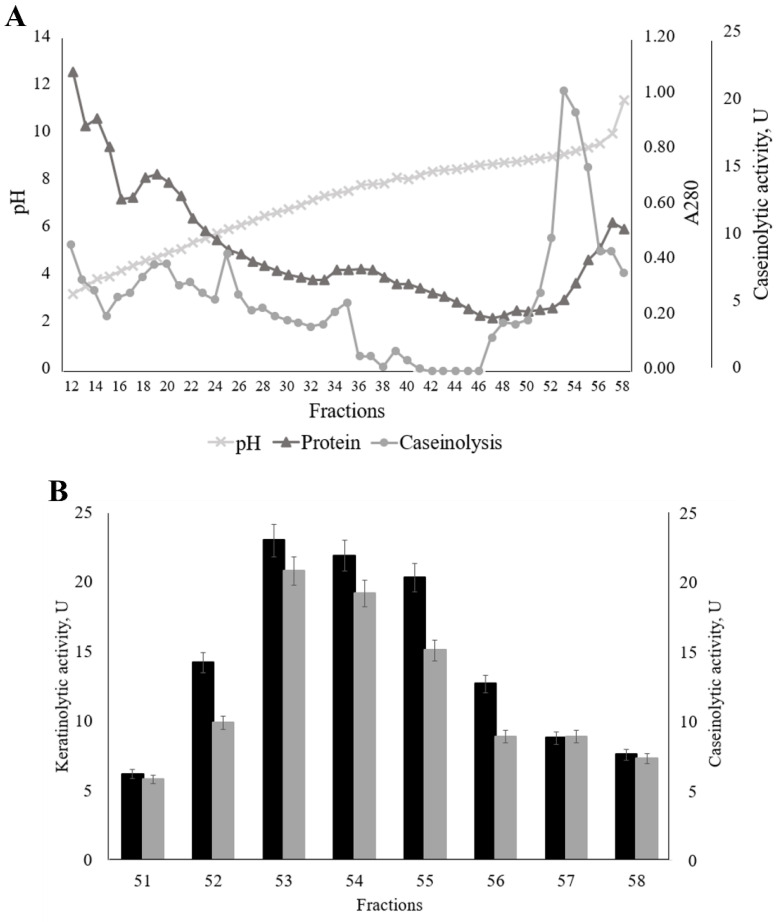
Isoelectric focusing of the extracellular proteins synthesized by *Aspergillus clavatus* VKPM F-1593 in the ampholines’ pH range from 3.0 to 10.0: (**A**) full fraction profiles of pH, protein content, and caseinolytic activity; (**B**) keratinolytic (black) and caseinolytic (grey) activity in the fractions with the highest level of caseinolysis.

We obtained an extracellular protein complex preparation by salting the culture liquid proteins with ammonium sulfate on the 4th day of the *A. clavatus* VKPM F-1593 submerged fermentation for further research. The precipitated light brown proteins were dissolved in the buffer, dialyzed, and lyophilized. The proteins of the resulting lyophilized preparation (pale brown powder) were separated by isoelectric focusing in a column with a sucrose density gradient of 0–40% and an ampholines’ pH range of 3.0–10.0. The pH, protein content, and caseinolytic activity were measured in the fractions after isoelectric focusing (Figure 5A). Keratinolytic activity was determined in the fractions with the highest values of caseinolytic activity (Figure 5B).

**Figure 6:**
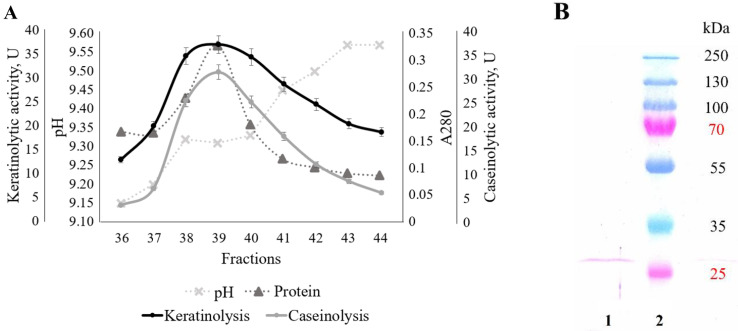
Isoelectric focusing of extracellular proteins synthesized by *Aspergillus clavatus* VKPM F-1593 in the ampholines’ pH range from 8.0 to 10.5: (**A**) fraction profiles of pH, protein amount, keratinolytic and caseinolytic activity; (**B**) SDS-PAGE of extracellular keratinase (fraction 39 (1); pre-stained protein ladder (2)).

The presence of keratinolytic activity was shown in the pH 9.0–11.5 samples. The highest value of protease activity (keratinolytic, 23.0 U; caseinolytic, 20.8 U) corresponded to pH 9.2. In this regard, we also conducted isoelectric focusing in the narrow pH range of ampholines (8.0–10.5) (Figure 6A). In addition, we performed electrophoresis (SDS-PAGE) to confirm the purity of the enzyme in the fractions (Figure 6B).

**Figure 7:**
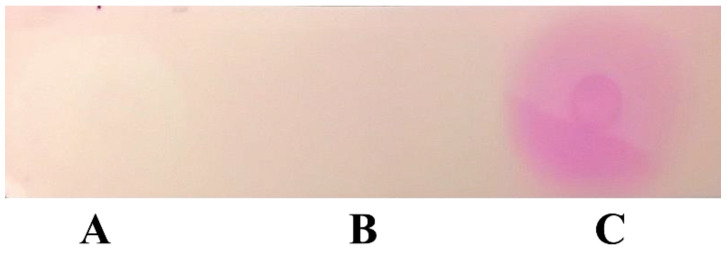
Result of the qualitative reaction to glycoproteins: bovine serum albumin (**A**); *Aspergillus clavatus* VKPM F-1593 extracellular keratinase (**B**); invertase (**C**).

Complex preparation proteins separated better in the second experiment due to IEF in the narrow pH range of ampholines (8.0–10.5). The peak keratinolytic (36.6 U) and caseinolytic (30.9 U) activity corresponded to the pH 9.3 fractions, which were also the samples with the maximum protein content. The molecular weight of the studied enzyme is about 27 kDa. We also conducted a qualitative reaction for glycoproteins with the keratinolytic protease, thereby revealing that keratinase was not glycosylated (Figure 7).

### 3.4. Substrate Specificity and Inhibitory Assay of the Aspergillus Clavatus VKPM F-1593 Extracellular Keratinase

**Figure 8:**
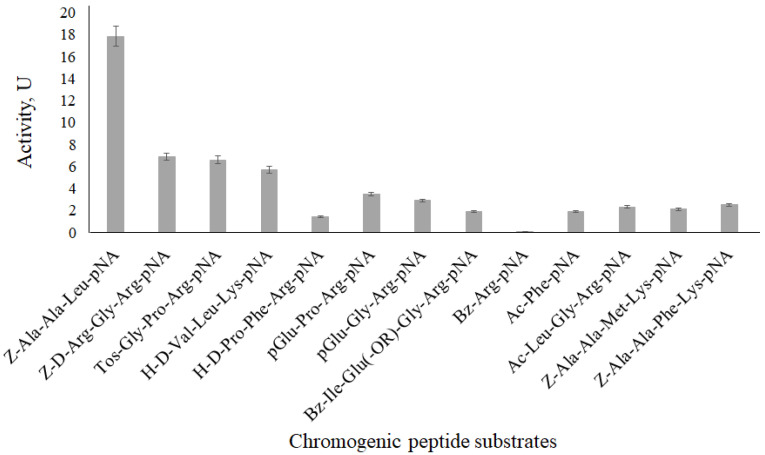
Substrate specificity of the extracellular keratinase synthesized by *Aspergillus clavatus* VKPM F-1593.

Substrate specificity of the *A. clavatus* VKPM F-1593 extracellular keratinase (pI 9.3) was studied using CPSs (Figure 8). The protease secreted by *A. clavatus* VKPM F-1593 showed the highest activity (17.85 U) with a chromogenic peptide substrate, which includes aliphatic uncharged amino acid residues—Z–Ala–Ala–Leu–pNA (ZAALNA). ZAALNA was applied for the keratinase inhibitory assay.

We investigated the effect of the following protease inhibitors on the activity of the studied enzyme: EDTA (metalloprotease inhibitor); PCMB (cysteine protease inhibitor); PMSF (serine protease inhibitor); TPCK (inhibitor of chymotrypsin-like proteases); TLCK and SBTI (trypsin-like protease inhibitors). Enzyme activity without preincubation with inhibitors was taken as 100%.

**Figure 9:**
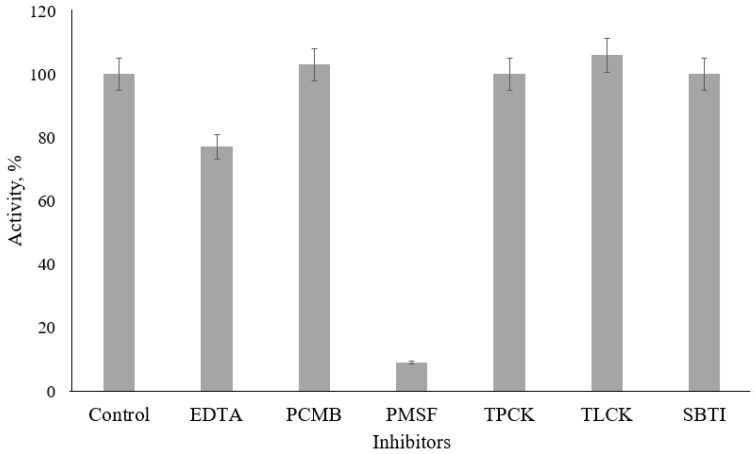
Effects of protease inhibitors on enzyme activity of the extracellular keratinase synthesized by *Aspergillus clavatus* VKPM F-1593.

Only two inhibitors that were used had a suppressive effect on the proteolytic activity of the keratinase against ZAALNA. PMSF inhibited enzyme activity by about 90%. Furthermore, the application of EDTA led to a decrease in activity by about 25% (Figure 9).

### 3.5. Temperature and pH Effect on the Activity and Stability of the Aspergillus clavatus VKPM F-1593 Extracellular Keratinase

**Figure 10:**
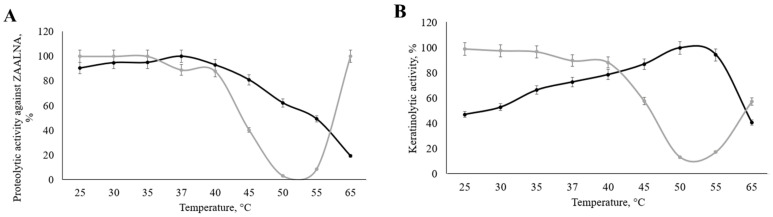
Activity of the *Aspergillus clavatus* VKPM F-1593 keratinase against ZAALNA (**A**) and keratin (**B**) dependency on temperature: T-optimum (black lines); T-stability (grey lines).

**Figure 11:**
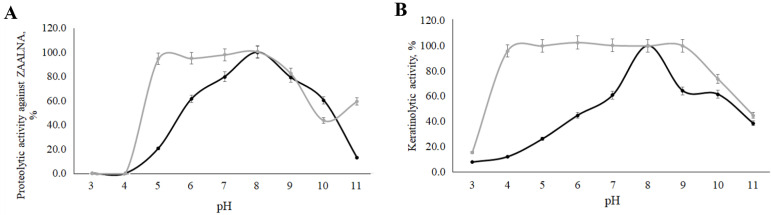
Activity of the *Aspergillus clavatus* VKPM F-1593 keratinase against ZAALNA (**A**) and keratin (**B**) dependency on pH: pH-optimum (black lines); pH-stability (grey lines).

We examined the dependence of the studied keratinase’s proteolytic activity and stability on pH and temperature (Figure 10 and Figure 11). The maximum activity value was taken as 100%.

When determining the dependence of enzymatic activity on the reaction temperature, the highest level of activity against ZAALNA corresponded to 37 °C, while the peak keratinolytic activity was recorded at 50 °C. At least 50% of the activity is retained at 25–55 °C in the studied temperature range, both during keratinolysis and ZAALNA hydrolysis. The enzyme sustained about 90% or more of its stability at 25–40 °C; the protease almost completely lost stability at 50 °C.

The maximum proteolytic activity corresponded to pH 8.0, both for reactions with keratin and with ZAALNA. Proteolytic activity remained above 60% in the pH range from 7 to 10. The stability of the enzyme persisted above 70% at pH 4–10 during keratinolysis, while the stability decreased more strongly and prevailed only at the level of 0% at pH 4 and 45% at pH 10 during ZAALNA hydrolysis.

## 4. Discussion

Micromycetes are well-known producers of proteolytic enzymes, including the ones active against fibrillar proteins [30]. The last decade of research on fungal keratinases has been focused on non-dermatophyte producers [39,40,41,42,43,44]. Representatives of genus *Aspergillus* are among the main research objects in this area. The four studied strains (*Aspergillus clavatus* VKPM F-1593, *A. amstelodami* A6, *A. ochraceus* 247, and *Cladosporium sphaerospermum* 1779) proved to be promising keratinolytic agents for biodegradation at the screening stage on agar media, not only because of high enzymatic indices on the media with keratin and casein, but also due to the low ability to hydrolyze elastin, since elastolytic activity is often determined as a pathogenicity factor [45,46,47].

**Figure 12:**
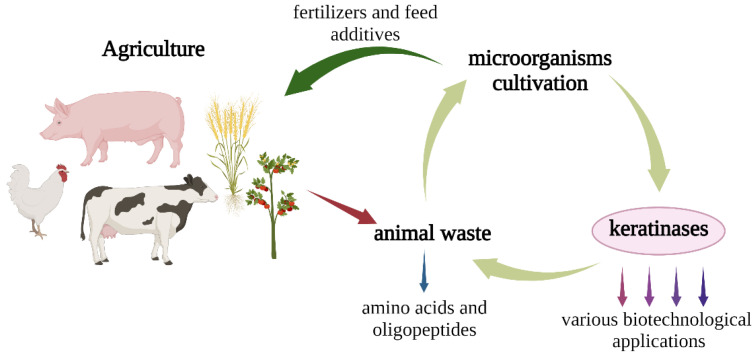
The scheme of closed-loop agriculture using keratinolytic microorganisms for animal waste biodegradation (created with BioRender.com, access date: 9 November 2021).

Constitutive synthesis is often considered as a biotechnological advantage in applied studies because usually enzyme substrates are expensive components. Nevertheless, it should be taken into account that in the field of biodegradation, the inductor is a waste, the use of which as a substrate for producer growth will not only reduce the cost of obtaining the target protease, but also lead to agricultural byproduct disposal both at the enzyme preparation step and during acquisition (Figure 12). Consequently, keratinolytic enzyme secretion by *Aspergillus clavatus* VKPM F-1593 during submerged fermentation in the presence of milled chicken feathers should be viewed as a positive characteristic.

The capacity of *Aspergillus* strains to synthesize keratinolytic enzymes is widely known (Table 3). However, some micromycete species belonging to this genus can cause aspergillosis [48]. At the same time, many *Aspergillus* fungi are already used in biotechnological industries as producers of valuable substances, including enzymes, and are recognized as corresponding to the GRAS status [49,50]. Genus *Aspergillus* contains several sections. Until recently, representatives of section *Clavati* had not been described as a potential keratinase source. In 2022, Qiu et al., published data on a new *Aspergillus clavatus* metalloprotease with keratinolytic activity (AcMep), [51]. Our study expands understanding of this filamentous fungus’ capability to hydrolyze keratin. The enzyme described by us is apparently a serine subtilisin-like protease, as indicated by the results of inhibitory and substrate analyses. The new keratinase has a lower molecular weight than the previously specified metalloprotease (27 and 44 kDa, respectively). In contradistinction to recombinant keratinase AcMep, the serine protease is not glycosylated, which increases the number of potential expression hosts for future research.

The known *Aspergillus* keratinases are mainly alkaline serine proteases with optimum pH at 8 and a temperature optimum at 50 °C (Table 4). A new serine keratinase with an isoelectric point of 9.3 showed the same properties. This protease was not highly thermostable but remained stable over a wide pH range. The studied features of the *Aspergillus clavatus* serine protease indicate the prospects of this producer and the necessity for further research.

The low cost of enzymes and their pH stability are important for animal waste biodegradation. The new serine protease secreted by *Aspergillus clavatus* VKPM F-1593 meets these parameters due to the producer growth on cheap substrates (milled chicken feathers) and the manifestation of high keratinolytic activity in the pH range of 4–10. Two-stage processing of poultry byproducts based on the *Aspergillus clavatus* VKPM F-1593 use will most likely be possible by micromycete cultivation and by the application of an enzyme preparation containing the keratinase. The first stage should be considered as an effective way to reduce the amount of waste due to the high growth rate of the micromycete. The production of keratinolytic enzymes, which are in demand in biodegradation and various industries, will also be carried out at this stage. The second one will be aimed at obtaining oligopeptides and amino acids. More research and scaling up are needed to understand the effectiveness of these processes and their cost-effectiveness. However, it can already be noted that this method is promising in comparison with the current approaches to animal waste disposal (landfilling and incineration) since biodegradation does not require huge areas, does not lead to the development of pathogenic microorganisms, and does not cause the appearance of new pollutants.

## 5. Conclusions

*Aspergillus clavatus* is a micromycete with a recently discovered capacity to secrete keratinases. To the already existing data on the micromycete’s ability to synthesize keratinolytic metalloproteases, within the framework of this research, knowledge on the possibility of obtaining subtilisin-like pH-stable keratinase was added. The producer and its keratinase properties indicate the promise of their application in the biodegradation field.

## Figures and Tables

**Table 1:** Modified Czapek medium composition.

Components	Medium 1, %	Medium 2, %	Medium 3, %
K_2_HPO_4_	0.1		
MgSO_4_ × 7H_2_O	0.05		
KCl	0.025		
NaCl	0.025		
FeSO_4_ × 7H_2_O	0.001		
Glucose	3.0		
Keratin	0.5	0.5	–
NaNO_3_	–	0.3	0.3

**Table 2:** NCBI accession numbers for the sequences used for phylogenetic analysis.

Strain	LSU rRNA	*benA*
*Aspergillus clavatus* VKPM F-1593 (A16)	OK559552.1	OP254188
*Aspergillus clavatus* CBS 513.65	NG_070579.1	EU076340.1
*Aspergillus clavatus* CBS 514.65	MH870335.1	EU076339.1
*Aspergillus clavatus* NRRL 2254	U28392.1	EF669809.1
*Aspergillus clavatus* NRRL 1	U28390.1	EF669802.1
*Aspergillus clavatonanicus* NRRL 4741	U28397.1	EF669842.1
*Aspergillus felis* CBS 130245	NG_070010.1	MN969363.1
*Aspergillus fumigatus* CBS 386.75	MH872681.1	AY685168.1
*Aspergillus giganteus* NRRL 10	U28395.1	EF669789.1
*Aspergillus giganteus* CBS 101.64	MH869999.1	EU076337.1
*Aspergillus giganteus* CBS 117.56	MH869070.1	EU076336.1
*Aspergillus giganteus* CBS 526.65	NG_069722.1	EU076331.1
*Aspergillus longivesica* NRRL 5215	U28400.1	EF669847.1
*Aspergillus niger* ATCC 16888	NG_055744.1	FJ608394.1
*Aspergillus rhizopodus* CBS 450.75	MH872694.1	EU076327.1
*Neocarpenteles acanthosporus* CBS 558.71	AB003809.1	EU076322.1

**Table 3:** Ratios of enzymatic indices of *A. amstelodami* A6, *A. clavatus* VKPM F-1593, *A. ochraceus* 247, and *Cladosporium sphaerospermum* 1779.

Fungi	EI_ker._/EI_cas._	EI_gel./_EI_cas._	EI_fibr./_EI_cas._	EI_el._/EI_cas._
*A. amstelodami* A6	1.26	0.93	1.28	0.70
*A. clavatus* VKPM F-1593	0.71	1.13	0.75	0.58
*A. ochraceus* 247	0.97	1.37	1.05	0.90
*C. sphaerospermum* 1779	0.61	1.18	0.72	0.67

**Table 4:** Properties of some known *Aspergillus* keratinases.

Producers	Molecular Weight	pH-Optimum (Stability)	T-Optimum (Stability), °C	Type of Proteasse	References
*A. clavatus*	44	8	45	metallo	[51]
	27	8 (4–10)	50 (25–40)	serine	Data obtained in this study
*A. flavus*	31	8 (7–10)	45 (30–70)	serine	[52]
*A. fumigatus*	24	6 (4–8)	50	–	[53]
*A. niger*	60	–	–	serine	[54]
	130	–	–	serine	[54]
*A. oryzae*	60	8	50	metallo	[55]
*A. parasiticus*	36	7	50	serine	[40]
*A. stelliformis*	–	8	50	–	[44]
*A. sulphureus*	–	10 (6.5–9)	35 (25–60)	–	[56]
*A. sydowii*	–	8	50	–	[44]
*A. tamarii*	–	8 (5–11)	40 (10–40)	serine	[57]

## Data Availability

The data presented in this study are available on request from the corresponding author.
